# Termite mound soil based potting media: a better approach towards sustainable agriculture

**DOI:** 10.3389/fmicb.2024.1387434

**Published:** 2024-07-01

**Authors:** Shiney Kathbaruah, Badal Bhattacharyya, Shimantini Borkataki, Bhabesh Gogoi, Preeti Hatibarua, Sailen Gogoi, K. Sindhura Bhairavi, Pranab Dutta

**Affiliations:** ^1^Assam Agricultural University, Jorhat, India; ^2^CPGSAS, Central Agricultural University (Imphal), Umiam, India

**Keywords:** biological, correlation, physico-chemical, potting media, reproductive growth parameters, termite mound soil, vegetative growth parameters

## Abstract

Termite mound soils are known to possess unique physico-chemical and biochemical properties, making them highly fertile. Considering their rich nutrient content, the objective of the current experiment is to assess the physico-chemical properties and enzyme activities of termite mound based potting media and evaluate theirperformance for further exploration in floriculture. Potting media consisting of termite mound soil (TS) of a subterranean termite, *Odontotermes obesus* were prepared in 7 different combinations with garden soil (GS), sand (S) and farmyard manure (FYM) and a control (without termite mound soil), i.e., T_1_ (TS, GS, S, FYM (v:v:v:v /1:2:1:1)), T_2_ (TS, GS, S, FYM (v:v:v:v / 2:1:1:1)), T_3_ (TS, S, FYM (v:v:v / 2:1:1)), T_4_ (TS, GS, FYM (v:v:v / 2:1:1)), T_5_ (TS, GS, S (v:v:v / 2:1:1)), T_6_ (TS, S, FYM (v:v:v / 3:1:1)), T_7_ (TS, S, FYM (v:v:v / 1:1:2)) and control (GS, S, FYM (v:v:v / 2:1:1)). The samples were then analysed in laboratory. Experimental analysis on physico-chemical and biological parameters revealed superiority of T_7_ (TS, S, FYM (v:v:v / 1:1:2)) in terms of pH (7.15), organic carbon (2.13%), available nitrogen (526.02 kg ha^−1^), available phosphorus (56.60 kg ha^−1^), available potassium (708.19 kg ha^−1^), dehydrogenase activity (18.21 μg TTF g^−1^ soil day^−1^), Phosphomonoesterase (PME) activity (46.68 54 μg p-nitrophenol/gsoil/h) and urease activity (3.39 μg NH_4_-N g^−1^ soil h^−1^). Whereas T_4_ (TS, GS, FYM (v:v:v /2:1:1)) registered superiority in terms of PME activity (50.54 μg p-nitrophenol/gsoil/h), Fluorescein diacetate (FDA) activity (11.01 μgfluorescein/gsoil/h) and Soil Microbial Biomass Carbon (SMBC) (262.25 μg/g). Subsequent to the laboratory analysis, two best potting mixtures (T_7_ & T_4_) were selected and their performance was assessed by growing a test crop, *Tagetes erecta* cv. Inca Orange. Considering the growth parameters, the potting media: T_7_ was found to be significantly superior in terms of plant spread (39.64 cm), leaf area index (4.07), fresh weight (37.72 g), yield (317.81 g/plant), and diameter (9.38 cm) of flower over T_4_ & control. The Benefit:Cost (B:C) ratio meaning the ratio of net returns to total cost of cultivation was determined. The B:C ratio of raising marigold flower as potted plant in T_7_ was 1.10 whereas the B:C ratio of the potting mixture of T_7_ was 2.52. This shows that T_7_ potting media is also economically viable choice for commercial purposes.

## Introduction

Termite mounds are structures of soil built by termites in several tropical ecosystems (Jouquet et al., 2015). These biogenic structures are built to protect the colonies from various environmental and biological factors (Eze et al., 2020). These structures contain soil particles of clay and organic matter cemented together (Harit et al., 2017; Jouquet et al., 2021, 2022). Subterranean mound-building termite species *Odontotermesobesus* (Rambur) are mostly found concentrated in tropical regions of the world, viz., Africa, Asia, etc. (Ashraf et al., 2020). It is the most abundant species with mound constructing ability to be found in Assam (Biswas and Deka, 2019). The type of soil and climatic conditions favour the population growth of termites (Bhattacharyya et al., 2014). Mounds of *O. obesus* are conical while some can be cathedral or lenticular as in South India (Harit and Jouquet, 2021). Despite termites being “silent destroyers” (Bhairavi et al., 2021), they are key soil bioturbators in the tropics (Cheik et al., 2022) and their constructed mounds are highly beneficial with rich nutrient content and beneficial microorganisms. Apart from these, termite soil also contains some useful plant growth promoting bacteria such as *Bacillus* sp., *Citrobacter freundii*, *Azotobacter* sp., and *Pseudomonas* sp., which are capable of solubilizing phosphate and potassium (Adebajo et al., 2021). Such beneficial properties and microbial activity present in termite mounds makes it ideal for use as a potting mixture.

Due to termite activity, the mounds have some unique physico-chemical and biochemical properties as compared to the surrounding soils for e.g. pH, organic carbon, cation exchange capacity, NPK content, etc. The effects of termite mound soil on crop growth have been studied by many workers in paddy (Miyagawa et al., 2011), maize (Bama and Ravindran, 2018), and tomato (Garba et al., 2011), due to its ability to supply nutrients. Such experiments showed excellent growth effect of termite mound soil on aforementioned crops. But it has not been examined on floriculture till date. Moreover, its effect particularly as potting media had not been experimented. Hence, the present investigation has been credited as a maiden attempt from India to assess the effectiveness of termite mound soil as potting media.

Considering all the beneficial properties of termite mound soil, the aim of the current experiment is to investigate the influence of termite mound soil on conventional potting media by analyzing its physico-chemical and biological properties and then evaluate its effect on growth of floriculture plants like marigold. In India, marigold (*Tagetes* spp.) occupies about two-third of the total area under cultivation with a total production of 1,754,000 MT, from an area of 255,000 ha (Kaur et al., 2021).

## Materials and methods

### Experimental site

The present experiment was carried out in the campus of Assam Agricultural University, Jorhat, Assam, India. It is situated in 26^°^72/N latitude and 94^°^20/ E longitude, at an altitude of 86.6 m above mean sea level. The mean annual rainfall is 2,375 mm while the maximum and minimum temperature are 33.9 and 9.7°C, respectively. The soil type is alluvial and vegetation includes wide variety from wild plant species to cultivated crops.

### Experimental materials

The top portion of the soil of randomly selected five termite mounds of *O. obesus* was collected (10kgs) and mixed up thoroughly to prepare a composite sample. The collected soil was spread on plastic sheets and was exposed to sunlight for 2 days for the escape of live termites. The garden soil, sand and FYM was collected from the experimental site. All the components were then thoroughly mixed according to the treatments and samples were collected for laboratory analysis. High quality hybrid seeds of African marigold (*Tagetes erecta*) variety Inca Orange were procured from “Syngenta flowers” and plastic pots (30 cm diameter) were used to grow the test crop. In the present study, the cultivar ‘Inca Orange’ was selected as it has remarkable growth characteristics and is fully adapted to agro-climatic conditions of Assam. Additionally, it has bagged the Royal Horticultural Society’s Award of Garden Merit.

### Preparation of potting media

The potting media was prepared using termite mound soil, garden soil, sand and FYM (Farmyard manure), i.e., decomposed mixture of cow litter and urine in different proportions.

### Treatments

The treatments used were as follows: T_1_—Termite mound soil, garden soil, sand and FYM at 1:2:1:1, T_2_—Termite mound soil, garden soil, sand and FYM at 2:1:1:1, T_3_—Termite mound soil, sand and FYM at 2:1:1, T_4_—Termite mound soil, garden soil and FYM at 2:1:1, T_5_—Termite mound soil, garden soil and sand at 2:1:1, T_6_—Termite mound soil, sand and FYM at 3:1:1, T_7_—Termite mound soil, sand and FYM at 1:1:2 and control-garden soil, sand and FYM at 2:1:1. Each of these treatments including control were replicated thrice.

### Processing of potting media for laboratory analysis

Potting media was prepared as per the treatment combinations and were dried, clods were broken down and the dried soil was sieved using a 2 mm sieve for laboratory analysis. Before drying, one part of each combination was left fresh and moist at 4°C without any processing to analyze the enzymatic properties of soil.

### Analysis of potting media samples

Soil physico-chemical properties such as pH, electrical conductivity, water holding capacity, infiltration rate, organic carbon, available nitrogen (N), available phosphorus (P) and available potassium (K) and biological properties such as soil microbial biomass carbon (SMBC) and enzyme activities viz., dehydrogenase, phosphomonoesterase (PME), fluorescein diacetate (FDA), and urease were analysed.

The pH and EC, 1:2.5 were determined though soil water suspension method (Jackson, 1973) while Keen Raczkowski box method was utilized for water holding capacity and infiltration rate (Keen and Raczkowski, 1921). Techniques employed to measure the organic carbon, available N, P, and K include wet digestion method (Walkley and Black, 1934), alkaline permanganate method (Subbiah and Asija, 1956), Bray and Kurtz no.1 method (Jackson, 1973) and neutral N Ammonium acetate method (Jackson, 1973), respectively.

For estimation of biological properties, SMBC was determined by chloroform fumigation extraction method (Jenkinson and Powlson, 1976). The enzymatic activity of dehydrogenase was estimated according the methodology described by Casida et al. (1964), PME was measured using p-nitrophenol phosphate (Tabatabai and Bremner, 1969) and the technique utilized for urease was non-buffer method using colorimetric determination of ammonium (Kandeler and Gerber, 1988).

Out of the seven treatments, two best treatments were selected from the laboratory analysis and the test crop was grown in pots in those best treatments. Best treatment, i.e., high organic carbon, high available nitrogen, phosphorus, potassium, pH around neutral and high biological activity.

### Experimental design

The pot experiment was laid out in Completely Randomized Design (CRD) with 2 best treatments and a control with 8 replications under field conditions.

The seeds of the marigold variety Inca Orange were sown on 10-11-2022 and transplanted to the pots on 10-12-2022. Double pinching was performed. First pinching was carried out ten days after visibility of first flower bud and second pinching was done ten days after single pinching.

### Analysis of morphological growth characteristics of the test crop

Morphological characters viz., vegetative and reproductive growth characteristics were measured during the mature flowering stage of the plant. Vegetative growth parameters were—plant height, plant spread, number of branches per plant, number of leaves per plant, leaf area index (LAI), stem diameter, number of roots per plant, root length and root diameter. The reproductive growth parameters included days to visibility of first flower bud, days to opening of flower, days to full bloom, flowering duration on plant, days from opening to fading of individual flower, flower bud diameter, flower diameter, number of flowers per plant, fresh weight of flower and yield of flower.

After harvesting of the crop at the end of the season, the potting media from the crop root zone was collected and again analysed for physico-chemical and biological properties to see the effect of crop on potting media.

### Economics

The cost of cultivation was calculated out for each treatment per 100 pots. The labour and operational cost and all the prevailing inputs were taken into account while calculating the cost of cultivation. The value of products at the prevailing market price is the gross return while the net return is calculated by subtracting the cost of cultivation from gross return. The benefit cost (B:C) ratio was calculated by dividing the net return by cost of cultivation. The B:C ratio as a potted plant and the B:C ratio for potting media was calculated accordingly.

### Statistical analysis

The physico-chemical and enzymatic activities of potting media were statistically analysed and the quantitative data pertaining to various growth characteristics of test crop were statistically analysed adopting the procedure of Analysis of Variance (ANOVA) by Panse and Sukhatme (1985). Whenever variance ratio was found significant, critical difference (CD) was calculated at 5 per cent level of significance, otherwise only Standard Error of Difference (S.Ed) (±) was mentioned. Pearson’s correlation technique was used to measure the correlation among the physico-chemical and biological parameters of potting media. The analyses were carried out using version 21 of IBM SPSS.

## Results

### Physico-chemical properties of potting media

The outcome of the physico-chemical properties presented in Table 1 showed that the pH of the potting media ranged from 5.14–8.03. T_3_ (7.15), T_7_ (7.15), and T_4_ (6.95) were observed to be nearly neutral range ideal for growing the test crop. The highest EC (1.88 ds/m) and water holding capacity (32.46%) were observed in T_6_ while the least value (1.45 ds/m) was recorded in T_1_ and T_5_ which were statistically at par with control (1.47 ds/m). Highest water holding capacity (32.46%) was reported in T_6_ while the lowest (26.00%) was reported in control and the vice versa was observed in the infiltration rate. The infiltration rate was highest in control (9.39 mm/h) while lowest in T_6_ (5.34 mm/h). The organic carbon content of the potting media ranged from 0.85 to 2.13% (Table 1). It was found to be significantly high in T_7_ (2.13%) followed by T_4_ (1.29%) which was statistically at par with T_6_ (1.27%) while the lowest was recorded in T_5_ (0.85%). According to the data, maximum amount of available nitrogen was recorded in T_7_ (526.02 kg ha^−1^) followed by T_4_ (275.17 kg ha^−1^) while the minimum amount was found in T_5_ (162.97 kg ha^−1^). Available phosphorus content was observed to be significantly highest in T_7_ (56.60 kg ha^−1^) followed by T_4_ (34.31 kg ha^−1^) while lowest available phosphorus content was found to be in T_5_ (22.07 kg ha^−1^). The highest available potassium content was observed in T_7_ (708.19 kg ha^−1^), followed by T_4_ (621.54 kg ha^−1^) while the lowest was observed in T_5_ (308.25 kg ha^−1^) and control (308.25 kg ha^−1^).

**Table 1:** Physico-chemical properties, available nutrient content, and biological properties of termite mound soil based potting media.

Treatment combinations	Physico-chemical properties and available nutrient content								Biological properties				
	pH	EC (ds/m)	Water holding capacity (%)	Infiltration rate (mm/h)	Organic carbon (%)	Available nitrogen (kg ha^−1^)	Available phosphorus (kg ha^−1^)	Available potassium (kg ha^−1^)	SMBC (μg/g)	Dehydrogenase (μg TTF g^−1^ soil day^−1^)	PME (μg p-nitrophenol/g soil/h)	FDA (μg fluorescein/g soil/h)	Urease (μg NH_4_-N g^−1^ soil h^−1^)
T_1_	6.50 ± 0.07	1.45 ± 0.11	26.50 ± 0.46	8.60 ± 0.04	1.09 ± 0.00	224.00 ± 5.20	24.35 ± 1.03	506.04 ± 0.09	186.79 ± 0.46	4.88 ± 0.12	34.16 ± 4.28	5.06 ± 0.06	2.02 ± 0.03
T_2_	6.70 ± 0.49	1.58 ± 0.08	29.00 ± 0.30	8.44 ± 0.10	1.08 ± 0.02	243.00 ± 1.02	25.57 ± 0.18	561.29 ± 1.02	116.27 ± 1.09	5.08 ± 0.18	42.22 ± 1.69	5.25 ± 0.23	2.11 ± 0.10
T_3_	7.15 ± 0.05	1.80 ± 0.04	27.75 ± 1.11	8.18 ± 0.07	1.24 ± 0.08	227.00 ± 0.60	25.00 ± 0.36	576.38 ± 0.90	128.97 ± 0.07	9.15 ± 0.77	40.68 ± 1.61	6.27 ± 0.06	2.20 ± 0.02
T_4_	6.95 ± 0.03	1.77 ± 0.00	31.18 ± 0.98	7.79 ± 0.05	1.29 ± 0.20	275.17 ± 5.41	34.31 ± 0.06	621.54 ± 0.37	262.25 ± 1.17	12.64 ± 1.44	50.54 ± 1.55	11.01 ± 0.70	2.24 ± 0.03
T_5_	5.14 ± 0.03	1.45 ± 0.03	27.00 ± 0.27	6.26 ± 0.05	0.85 ± 0.04	162.97 ± 0.09	22.07 ± 0.99	308.25 ± 0.91	109.36 ± 0.95	2.64 ± 0.46	29.17 ± 4.39	3.66 ± 0.23	0.56 ± 0.02
T_6_	8.03 ± 0.01	1.88 ± 0.02	32.46 ± 0.98	5.34 ± 0.04	1.27 ± 0.03	243.90 ± 0.25	32.20 ± 1.02	612.33 ± 0.95	212.49 ± 0.95	3.83 ± 0.34	44.03 ± 1.70	6.3 ± 0.13	2.25 ± 0.08
T_7_	7.15 ± 0.23	1.74 ± 0.02	28.59 ± 0.95	8.58 ± 0.11	2.13 ± 0.02	526.02 ± 5.34	56.60 ± 1.11	708.19 ± 1.91	235.01 ± 0.21	18.21 ± 1.47	46.68 ± 2.23	8.18 ± 0.13	3.39 ± 0.05
Control	6.21 ± 0.17	1.47 ± 0.01	26.00 ± 0.66	9.39 ± 0.06	1.06 ± 0.06	213.58 ± 0.96	26.30 ± 0.80	308.25 ± 0.82	181.10 ± 1.72	9.45 ± 0.50	38.87 ± 3.37	7.05 ± 0.06	3.19 ± 0.01
S.Ed (±)	0.17	0.04	0.64	0.07	0.03	2.69	0.65	0.82	0.79	0.67	2.32	0.23	0.04
CD (*p* = 0.05)	0.29	0.07	1.11	0.13	0.05	4.69	1.13	4.42	1.38	1.17	4.05	0.40	0.07

Mean values ± standard deviation.SMBC, soil microbial biomass carbon; PME, phosphomonoesterase; FDA, fluorescein diacetate.

### Biological and enzymatic properties of potting media

The highest SMBC was recorded in T_4_ (262.25 μg/g) followed by T_7_ (235.01 μg/g) while the lowest microbial biomass carbon was observed in T_5_ (109.36 μg/g) (Table 1). In case of dehydrogenase activity, it was revealed that T_7_ had the highest dehydrogenase activity (18.21 μg TTF g^−1^ soil day^−1^) which was followed by T_4_ (12.64 μg TTF g^−1^ soil day^−1^). However, the lowest dehydrogenase activity was observed in T_5_ (2.64 μg TTF g^−1^ soil day^−1^). The highest PME activity was recorded in T_4_ (50.54 μg p-nitrophenol/g soil/h) which was found to be statistically at par with T_7_ (46.68 μg p-nitrophenol/g soil/h). The minimum PME activity was recorded in T_5_ (29.17 μg p-nitrophenol/g soil/h). T_4_ (11.01 μg fluorescein/g soil/h) had the highest amount of FDA activity which was followed by T_7_ (8.18 μg fluorescein/g soil/h). The lowest FDA activity was measured in T_5_ (3.66 μg fluorescein/g soil/h). Lastly, the highest urease activity was observed in T_7_ (3.39 μg NH_4_-N g^−1^ soil h^−1^) which was statistically at par with the control (3.19 μg NH_4_-N g^−1^ soil h^−1^). It was followed by T_4_ (2.24 μg NH_4_-N g^−1^ soil h^−1^) and T_3_ (2.20 μg NH_4_-N g^−1^ soil h^−1^) while the lowest was registered in T_5_ (0.56 μg NH_4_-N g^−1^ soil h^−1^).

### Correlation studies among physico-chemical and biological properties of potting media

Pearson correlation (Table 2) between the different parameters of physico-chemical and biological properties showed that soil pH had significant positive correlation with EC (*r* = 0.86, *p* < 0.01), water holding capacity (*r* = 0.72, *p* < 0.05), dehydrogenase (*r* = 0.71, *p* < 0.05) and PME activity (*r* = 0.74, *p* < 0.05). Similarly, EC showed significant positive correlation with water holding capacity (*r* = 0.80, *p* < 0.05), dehydrogenase (*r* = 0.73, *p* < 0.05) and PME activity (*r* = 0.76, *p* < 0.05). The water holding capacity was positively correlated with PME activity (*r* = 0.72, *p* < 0.05). Organic carbon had significant positive correlation with available phosphorus (*r* = 0.97, *p* < 0.01), available potassium (*r* = 0.94, *p* < 0.01), and dehydrogenase activity (*r* = 0.86, *p* < 0.01). Available nitrogen had significant positive correlation with urease activity (*r* = 0.87, *p* < 0.01). Available phosphorus had significant positive relationship with available potassium (*r* = 0.90, *p* < 0.01), dehydrogenase (*r* = 0.87, *p* < 0.01), and PME activity (*r* = 0.73, *p* < 0.05). Available potassium displayed a significant positive relationship with dehydrogenase activity (*r* = 0.78, *p* < 0.05). SMBC was positively correlated with dehydrogenase (*r* = 0.78, *p* < 0.05), PME (*r* = 0.71, *p* < 0.05), FDA (*r* = 0.87, *p* < 0.01) and urease activity (*r* = 0.77, *p* < 0.05). Correlation analysis showed that dehydrogenase had significant positive correlation with PME (*r* = 0.80, *p* < 0.05), FDA (*r* = 0.71, *p* < 0.05) and urease activity (*r* = 0.74, *p* < 0.05). Significant positive correlation of PME activity was observed with FDA activity (*r* = 0.85, *p* < 0.01).

**Table 2:** Simple correlation among physico-chemical and biological properties of potting media.

Parameters	pH	EC	WHC	IR	OC	Avail N	Avail P_2_O_5_	Avail K_2_O	SMBC	DHA	PME	FDA	UREASE
pH	1.00												
EC	0.86^**^	1.00											
WHC	0.72^*^	0.80^*^	1.00										
IR	−0.16	−0.37	−0.57	1.00									
OC	0.52	0.52	0.25	0.20	1.00								
Avail N	0.14	0.01	−0.12	0.57	0.63	1.00							
Avail P_2_O_5_	0.44	0.47	0.31	0.12	0.97^**^	0.66	1.00						
Avail K_2_O	0.64	0.63	0.40	0.08	0.94^**^	0.38	0.90^**^	1.00					
SMBC	0.52	0.45	0.53	0.03	0.60	0.46	0.66	0.55	1.00				
DHA	0.71^*^	0.73^*^	0.56	0.00	0.86^**^	0.63	0.87^**^	0.78^*^	0.78^*^	1.00			
PME	0.74^*^	0.76^*^	0.72^*^	0.11	0.63	0.38	0.73*	0.69	0.71^*^	0.80^*^	1.00		
FDA	0.41	0.46	0.52	0.21	0.51	0.48	0.58	0.47	0.87^**^	0.71^*^	0.85^**^	1.00	
UREASE	0.54	0.30	0.11	0.58	0.68	0.87^**^	0.63	0.54	0.77*	0.74^*^	0.63	0.57	1.00

*Significance at level of 0.05 and **Significance at a level of 0.01.EC, electrical conductivity; WHC, water holding capacity; IR, infiltration rate; OC, organic carbon; N, nitrogen; P_2_O_5_, phosphorus; K_2_O, potassium; SMBC, soil microbial biomass carbon; DHA, dehydrogenase activity; FDA, fluorescein diacetate; PME, phosphomonoesterase activity.

Thus, the physico-chemical and biological analysis of all the seven treatments revealed that both T_7_ and T_4_ were superior in terms of pH, organic carbon, available nitrogen, available phosphorus, available potassium, dehydrogenase, PME, urease activity and PME, FDA, SMBC, respectively. Therefore, these two treatments were selected for growing the test crop *Tagetes erecta* cv. Inca Orange.

### Vegetative growth parameters of the test crop

Results showed no significant difference among the treatments in case of plant height (Table 3). The mean value recorded in T_7_, T_4_ and control were 38.95, 39.28, and 38.45 cm, respectively and were statistically at par. The plant spread (39.64 cm) and leaf area index (4.07) was found to be highest in T_7_ while the number of branches (14.0), number of leaves per plant (398.50), and stem diameter (1.63 cm) were observed to be high in both T_7_ and T_4_, being statistically at par. Again, number of roots (35.63), root length (21.54 cm) and root diameter (1.5 mm) were highest in T_7_.

**Table 3:** Effect of potting media on vegetative and reproductive growth parameters of *T. erecta* cv. Inca Orange.

Treatments	Vegetative growth parameters									Reproductive growth parameters									
	Plant height (cm)	Plant spread (cm)	No. of branches per plant	No. of leaves per plant	Leaf area index	Stem diameter (cm)	No. of roots per plant	Root length (cm)	Root diameter (mm)	Days to visibility of first flower bud	Days to opening of flower	Days to full bloom	Flowering duration (days)	Days to opening to fading of individual flower	Flower bud diameter (cm)	Flower diameter (cm)	No. of flowers per plant	Fresh weight of flower (g)	Yield of flower (g/plant)
T_7_	38.95 ± 4.38	39.64 ± 1.98	14.00 ± 2.07	398.50 ± 41.54	4.07 ± 0.48	1.63 ± 0.12	35.63 ± 2.97	21.54 ± 0.82	1.50 ± 0.12	40.13 ± 1.55	55.75 ± 1.98	91.25 ± 4.62	58.50 ± 2.56	49.63 ± 2.13	0.74 ± 0.08	9.38 ± 0.40	22.50 ± 3.59	37.72 ± 1.71	317.81 ± 57.53
T_4_	39.28 ± 2.30	37.39 ± 2.57	13.12 ± 1.96	375.88 ± 36.53	3.43 ± 3.43	1.57 ± 0.08	33.38 ± 2.33	20.66 ± 1.26	1.43 ± 0.05	40.75 ± 1.91	57.13 ± 2.23	89.88 ± 2.95	58.50 ± 2.20	49.13 ± 2.30	0.71 ± 0.10	9.01 ± 0.30	18.63 ± 5.61	30.21 ± 2.89	269.38 ± 58.10
Control	38.45 ± 2.13	27.81 ± 2.27	9.88 ± 1.73	251.88 ± 59.10	1.25 ± 1.25	1.28 ± 0.11	25.88 ± 3.83	18.06 ± 1.32	0.91 ± 0.26	43.13 ± 1.89	62.88 ± 4.09	109.13 ± 10.54	63.00 ± 2.67	43.50 ± 2.07	0.53 ± 0.05	6.74 ± 0.23	33.13 ± 5.41	23.51 ± 2.13	166.99 ± 19.56
S.Ed (±)	1.56	1.14	0.96	23.37	0.19	0.05	1.55	0.58	0.09	0.90	1.46	3.43	1.24	1.08	0.04	0.16	2.48	1.15	24.27
CD (*p* = 0.05)	N/A	1.97	1.67	40.22	0.33	0.09	2.67	0.93	0.15	1.54	2.51	5.60	2.14	1.87	0.07	0.27	4.26	1.98	41.77

Mean values ± standard deviation. T_7_, Termite mound soil, sand and FYM (v:v:v / 1:1:2); T_4_, Termite mound soil, garden soil and FYM (v:v:v / 2:1:1); Control, Garden soil, sand and FYM (v:v:v / 2:1:1).

### Reproductive growth parameters of the test crop

The number of days to visibility of first flower bud was found to be shortest in T_7_ (40.13 days) and T_4_ (40.75 days) which were statistically at par (Table 3). Concurrently, the earliest opening of flower was observed in T_7_ (55.75 days) and T_4_ (57.13 days). The shortest time to full bloom was observed in T_7_ (91.25 days) and T_4_ (89.88 days) both being statistically at par. No significant difference was observed between T_7_ (58.50 days) and T_4_ (58.50 days) regarding flowering duration. The highest number of days from opening to fading of individual flower was observed in T_7_ (49.63 days) and T_4_ (49.13 days) being statistically at par. The flower bud diameter was reported to be high in T_7_ (0.74 cm) which was statistically at par with T_4_ (0.71 cm). The largest diameter of flower was recorded in T_7_ (9.38 cm) while the smallest flower diameter was observed in control (6.74 cm). The highest number of flowers were produced by control (33.13). Similarly, the maximum fresh flower weight and highest yield of marigold was recorded in T_7_ at 37.72 gm and 317.81 gm/plant, respectively.

### Impact of crop *Tagetes erecta* cv. Inca Orange on soil properties

**Figure 1:**
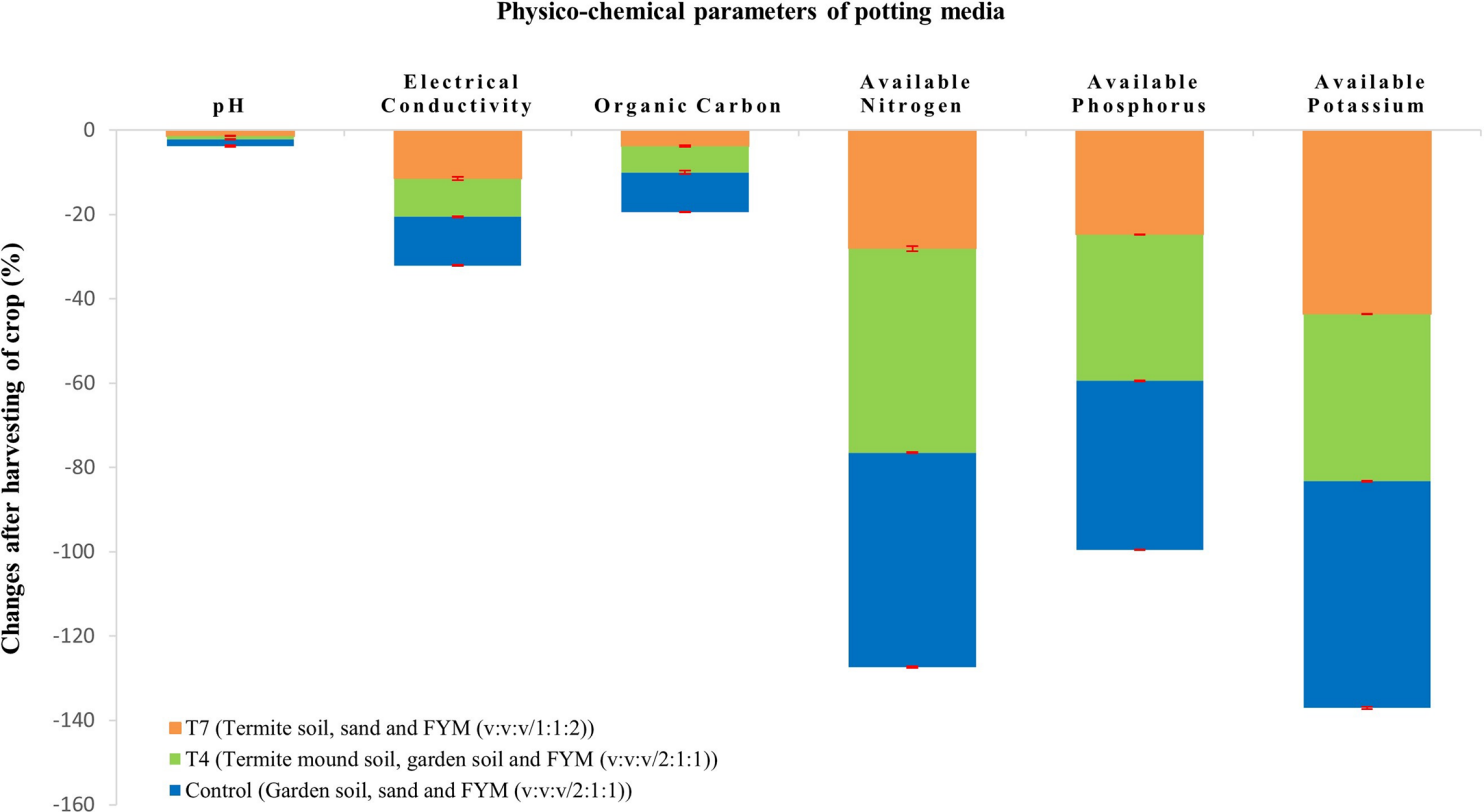
Impact of crop on physico-chemical parameters of potting media.

**Figure 2:**
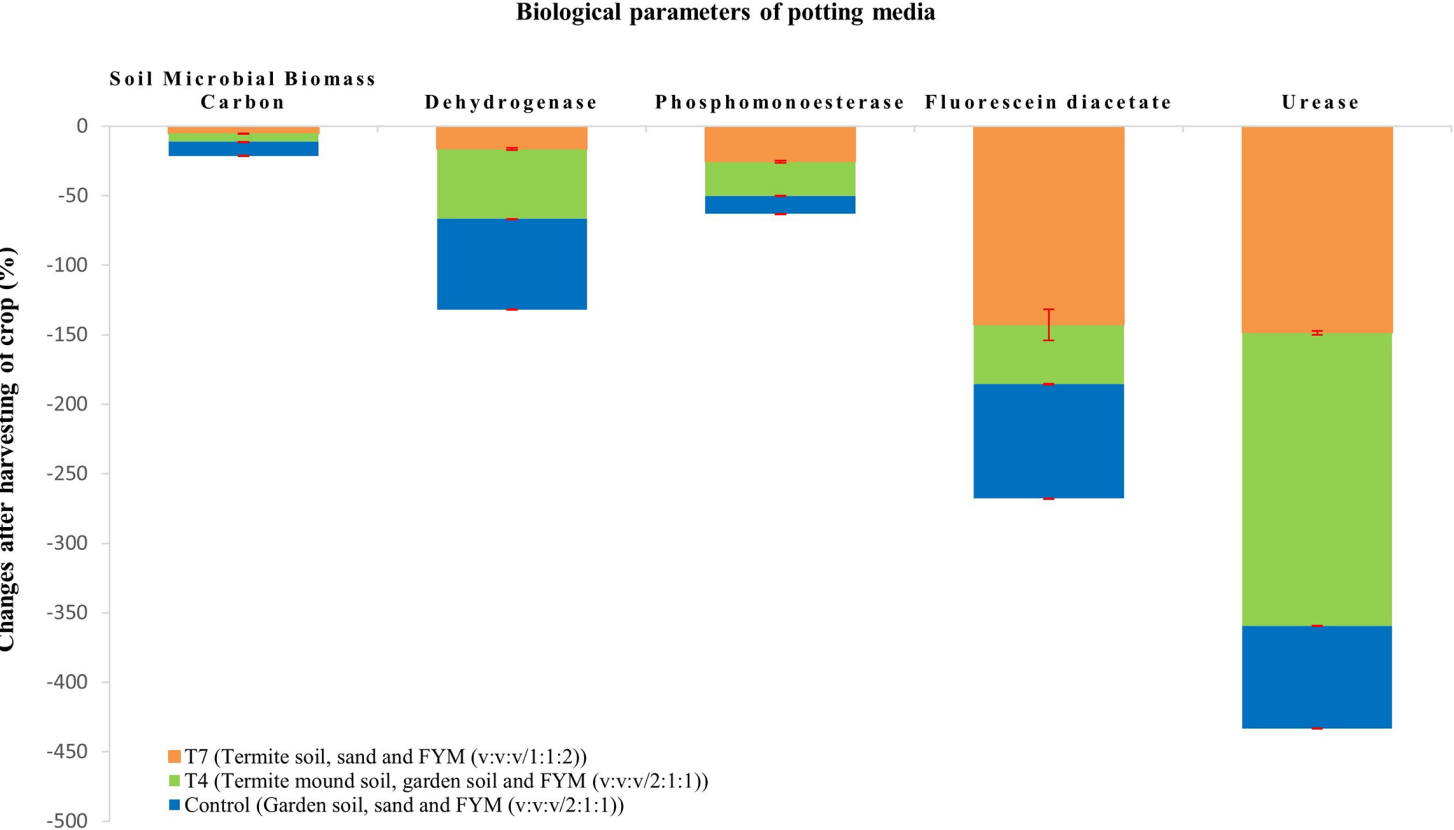
Impact of crop on biological parameters of potting media.

**Figure 3:**
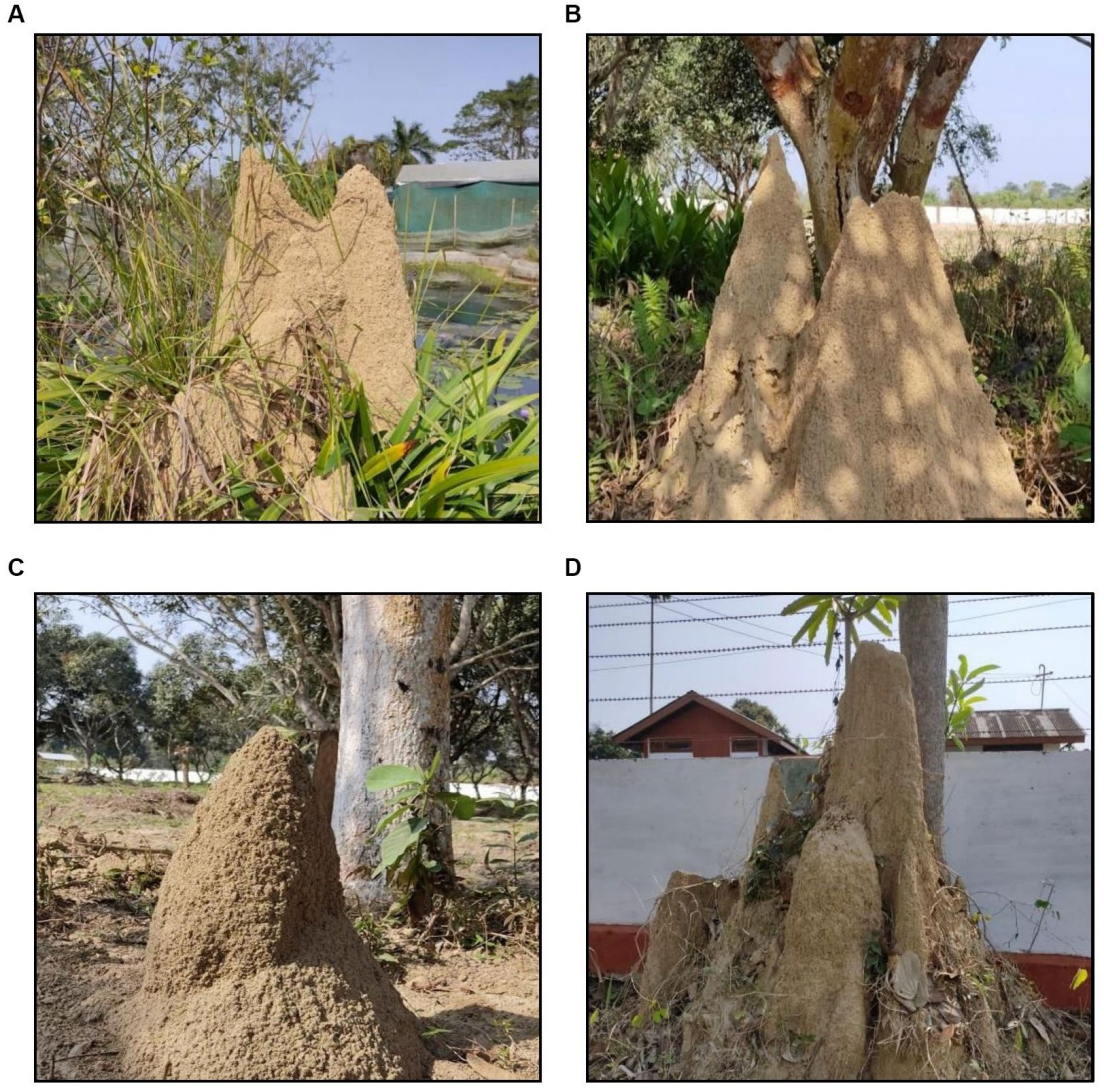
**(A–D)** Selected mounds of *O. obesus* for collection of termite mound soil.

At the end of the crop season, the crop was harvested and the potting media was tested to draw the impact of the crop on the potting media (Table 4). It is graphically depicted in Figures 1, 2. All the chemical properties viz., pH, EC, organic carbon, available nitrogen, available phosphorus and available potassium gradually decreased while all the biological properties viz., SMBC, dehydrogenase, FDA, PME and urease activity gradually increased after harvesting of the crop (Figures 3A–D).

**Table 4:** Comparison of potting media properties before and after growing crop.

Parameters	Before growing crop	After harvest of crop	Before growing crop	After harvest of crop
	T_4_		T_7_	
Physicochemical properties				
pH	6.95	6.90	7.15	7.05
EC (ds/m)	1.77	1.61	1.74	1.54
Organic Carbon (%)	1.29	1.21	2.13	2.05
Available Nitrogen (kg ha^−1^)	275.17	141.95	526.02	378.25
Available Phosphorus (kg ha^−1^)	34.31	22.40	56.60	42.59
Available Potassium (kg ha^−1^)	621.54	375.04	708.19	399.19
Biological properties				
Soil Microbial Biomass Carbon(μg/g)	262.25	278.32	235.01	246.91
Dehydrogenase (μg TTF g^−1^ soil day^−1^)	12.64	18.97	18.21	21.22
PME (μg p-nitrophenol/g soil/h)	50.54	62.95	46.68	58.64
FDA (μg fluorescein/g soil/h)	11.01	15.66	8.18	19.89
Urease (μg NH_4_-N g^−1^ soil h^−1^)	2.24	6.96	3.39	8.43

### Economics of *Tagetes erecta* cv. Inca Orange using termite mound based potting media

According to cost analysis (Table 5), the highest B:C ratio as a potted plant was observed in T_7_ (1.10). Similarly, the B:C ratio as a potting media (Table 5) was recorded to be highest in T_7_ (2.52).

**Table 5:** Economics of potted plants and potting media using termite mound soil.

Treatments	Economics of potting plants (per 100 pots)					Economics of potting media (per 100 pots)				
	Total cost of cultivation (Rs.) (A)	Sale price (Rs./pot)	Gross return (Rs.) (B)	Net return (Rs.) (B-A)	B: C Ratio	Total cost of cultivation (Rs.) (A)	Sale price (Rs./kg)	Gross return (Rs.) (B)	Net return (Rs.) (B-A)	B: C Ratio
T_7_	11,878.63	250	25,000	13,121.37	1.10	2,128.63	30	7,500	5,371.37	2.52
T_4_	11,427.94	230	23,000	11,572.06	1.01	2,277.94	25	6,250	3,972.06	1.74
Control	11,147.59	200	20,000	8,852.41	0.79	1,997.59	20	5,000	3,002.41	1.50

T_7_, Termite mound soil, sand and FYM (v:v:v / 1:1:2); T_4_, Termite mound soil, garden soil and FYM (v:v:v / 2:1:1); Control, Garden soil, sand and FYM (v:v:v / 2:1:1).

## Discussion

According to the data presented in Table 1, the pH values of the potting media were moderately acidic to moderately alkaline. Moderately acidic pH was observed only in T_5_ because of the lack of organic matter. Typically, termite mound soils have an acidic pH which explains the slight acidity of the treatments (Li et al., 2017; Apori et al., 2020). However, some other workers had reported pH of the termite mound soil as alkaline (Dhembare, 2013; Subi and Sheela, 2020). Contrary to the above information, some studies have reported no significant difference in the pH of termite mound soil and its adjacent soil (Brossard et al., 2007). The EC depends on the textural class (Hamarashid et al., 2010) and on the mineralisation of organic matter and presence of micronutrients. Therefore, highest EC in T_6_ was most likely due to highest proportion of termite mound soil. This result corroborates with the earlier work of Ibrahim et al. (2022) who reported higher EC in termite mound soil than the adjacent soil. The two major factors that affect the soil water holding capacity are soil organic matter and soil texture (Kartik and Jain, 2022). Fine textured soils like clay generally have a high water holding capacity as compared to other soil types. The composition of potting media in T_6_ and T_4_ registered higher organic matter and hence higher water holding capacity. The present study showed that the control, T_1_ and T_5_ had the lowest water holding capacity. Comparatively lower water holding capacities registered in the above treatments were probably because of low organic matter content of the potting mixtures_._ As a general fact, decaying plant material and termite excreta attributed to the high amount of organic carbon found in termite mound soils. A similar trend was reported by Bachha et al. (2022) who found 24.53 and 10.78 per cent water holding capacity in *Odontotermes* spp. mound soil and its surrounding soil, respectively, vividly indicating high water holding capacity in termite mounds. Infiltration rate determines how quickly water penetrates the soil and reaches the plant roots ensuring both water and nutrient availability to the crops. The potting media viz., T_1_ and T_7_ displayed relatively high infiltration rate which points to the availability of high amount of nitrogen, phosphorus and potassium. The present observations are in agreement with Adugna et al. (2016) and Ackerman et al. (2007) who noticed higher infiltration rate in termite mound than the adjacent soil due to termite foraging activity. Also, presence of FYM in enhancing soil infiltration rate was investigated by Zhang et al. (2014) who found that saturated hydraulic conductivity (a critical indicator of soil infiltration) had a significant effect due to application of FYM. The highest organic carbon was observed in T_7_ which was followed by T_4_. These two treatments contained relatively higher proportion of termite mound soil including FYM. Feeding of termites on cellulose along with its behaviour of gathering organic material for nesting purposes, promoting decomposition and nutrient cycling contributes greatly to high content of organic matter in soil. The possible reason for lowest organic carbon content as recorded in T_5_ might be due to the non-inclusion of FYM. Similar conclusions were drawn by Dhembare (2014) and Chisanga et al. (2020), who found higher organic carbon in termite mound soil than surrounding soil. High organic matter acts as a reservoir of nitrogen. Hence, high nitrogen content is present in termite mounds (Jose and Maya, 2020). Highest organic carbon in T_7_ can explain the high amount of available nitrogen in T_7_ due to organic matter mineralization. Moreover, the bacteria present in termite hindguts have proved to be an important pathway for nitrogen fixation as reported by Mullins et al. (2021). The potting media T_5_ recorded the lowest available nitrogen due to least amount of organic matter. Phosphorus availability is mostly affected by soil pH and organic matter. Highest available phosphorus recorded in T_7_ (Table 2) might be because of appropriate pH (7.15) and high organic matter content. In acid soils, phosphorus fixation takes place because of iron and aluminium oxides while in alkaline soils calcium and magnesium react with phosphorus which leads to phosphorus fixation. High available phosphorus in termite mounds as compared to surrounding soil was observed by Dhembare (2014) and Bachha et al. (2022). Organic matter plays a very important role in potassium availability though decomposition and mineralization processes. This was observed in case of T_7_ which had the highest available potassium content. Termite mound soils have high CEC which also explains relatively high availability of potassium in the potting media with mound soil. The present findings corroborate with that of Dhembare (2014) and Bachha et al. (2022) who found 10.08 and 0.37 per cent increase in potassium availability of mound soil as compared to surrounding soil, respectively. The potting mixture after harvesting of the crop showed a decline in organic carbon, available nitrogen, phosphorus and potassium (Figure 2). Lower available nitrogen might be due to plant uptake, leaching and volatilization. Similarly low available phosphorus could be attributed to plant uptake. Again, luxurious consumption of potassium by the test crop might seem to be the valid reason for low amount of available potassium. Organic carbon gradually decreased most likely due to organic matter mineralisation. This might also be the reason for decline in pH. The EC also had a gradual decrease due to depletion of micronutrients as a result of plant uptake.

Termite mound soil harbours soil microorganisms which play a vital role in organic matter decomposition and nutrient cycling which contributes greatly to microbial biomass carbon. High SMBC recorded in case of T_4_ and T_7_ was most likely due to the presence of beneficial microorganisms in termite mound soil. FYM also harbours a wide array of microorganisms indicating increased microbial biomass carbon content in case of T_7_. Issoufou et al. (2019) has demonstrated that termites significantly increase microbial biomass besides promoting significant activity of microbial decomposers leading to increase in degradation of soil organic matter. Dehydrogenases play a crucial role in the microbial respiration process and breakdown of organic matter. Due to presence of termite mound soil and relatively high amount of FYM, high dehydrogenase activity was observed in T_7_ and T_4_. More or less similar findings were reported by Subi and Sheela (2020). Lowest dehydrogenase activity was observed in T_5_, most probably because of absence of FYM. PME activity in potting media ranged from 29.17 to 50.54 μg p-nitrophenol/g soil/h. This group of enzymes play a vital role in the mineralization process and cycling of phosphorus in soil ecosystems. High PME activity was observed in T_4_ and T_7_ because of the high proportion of termite mound soil and FYM. Increase in enzyme activity due to increase in organic matter content was reported by Lee and Wood (1971). The present findings were in line with those of Roose-Amsaleg et al. (2005) who observed high phosphatase activity in fresh mound as compared to mature mound. FDA activity is commonly used as an indicator of total microbial activity in soil. High FDA was noticed in T_4_ preferably because of high microbial activity in termite mound soil. It was followed by T_7_ most likely due to the presence of relatively higher proportion of FYM. Lowest FDA activity was observed in T_5_ which could be attributed to the lack of organic matter. Urease is an enzyme that catalyses the hydrolysis of urea into ammonia and carbon dioxide. High urease activity was observed in T_7_ which might be attributed to the high organic matter and microbial present in termite mound soil. Low organic matter content can make enzyme prone to biological degradation as reported earlier by Baligar et al. (1991). Lowest urease activity was recorded in T_5_ probably due to the absence of organic matter. After harvesting of the crop at the end of crop season, biological activities seemed to increase (Figure 3). This might be attributed to the release of rhizodeposits and increase in microbial activity with time due to frequent irrigation and exposure to sunlight (Haldar and Sengupta, 2015).

Out of all the potting media treatments, T_7_ (Termite soil, sand and FYM (v:v:v / 1:1:2)) emerged out as the best one in terms of soil pH, organic carbon, available nitrogen, available phosphorus, available potassium, dehydrogenase enzyme activity, PME activity and urease activity while T_4_ (Termite soil, garden soil and FYM (v:v:v / 2:1:1)) showed superiority with regards to PME activity, FDA activity and SMBC.

In case of vegetative growth parameters of the test crop, double pinching might have caused the suppression of apical growth and resulted in similar heights in all the three treatments. The results were in close agreement with Pant et al. (2022) who found double pinching to be the factor for minimum plant height in African Marigold. Highest plant spread was observed in T_7_ while the lowest was observed in control (Table 3). High proportion of termite soil and presence of FYM might be the reason for the higher plant spread in case of T_7_. High availability of NPK, water holding capacity, organic matter content, good drainage and water retention were prevalent in T_7_. Highest numbers of branches (Table 3) were observed in T_7_ which was statistically at par with T_4_. The lowest number was observed in control. Prevalence of adequate nutrient levels in potting media due to combined effect of termite soil and FYM and double pinching performed might have led to activation of lateral buds. Meena et al. (2015) and Khobragade et al. (2012) also reported that there was development of more side branches after pinching. T_7_ and T_4_ were statistically at par and had high number of leaves as compared to control. The number of leaves corresponded with the number of branches as more branching ensures a greater number of leaves. Supportive evidence regarding the number of leaves was reported by Singh et al. (2017) and Singh et al. (2015). Highest leaf area index was observed in T_7_while the lowest was observed in control. High leaf area index in T_7_might be because of good branching habit, more number of leaves, high specific leaf area and plant density. The findings of Yadav et al. (2018) was found to be similar with the current study and found the leaf area index to be 1.14 in African marigold. T_7_ and T_4_ recorded high stem diameters while control had the lowest (Table 3). Increased nutrient availability can increase plant growth including stem diameter which was easily and adequately available in both T_7_ and T_4_ treatments. A comparatively low nutrient content, water holding capacity and infiltration rate in addition to alkaline nature of potting media in control treatment may have contributed to significantly low stem diameter. Present findings were in conformity with Pant et al. (2022). The highest number of roots (statistically at par with each other) were observed inT_7_ and T_4_ while control recorded the lowest number of roots (Table 3). A corresponding trend was observed in case of root length and number of roots, i.e., T_7_ and T_4_ were statistically at par while control recorded the lowest root length. Presence of high proportion of termite soil and FYM in the selected potting media accounts for high nutrient availability, crumbly and porous soil structure to promote aeration and water drainage and proliferation of beneficial soil microorganisms. The treatments, T_7_ and T_4_ possess elevated microbial activity which might have enhanced root growth. Termites make the soil porous accounting for better root growth (Aparna et al., 2021).

The first flower bud was observed in T_7_ and T_4_ potting media (statistically at par) and in control after 43.13 days (Table 3). The probable cause of early bud initiation may be due to rise in temperatures during the initiation of the reproductive phase. Light exposure in the field condition with ample sunshine hours and moisture with adequate nutrients from mixture of termite soil and FYM might have contributed to early initiation of flower bud. Sheoran et al. (2022) observed a similar result at 40.66 days. The delay in days to flower opening might have been influenced by double pinching. The delay in days to flower opening might have been influenced by double pinching. It can be assumed that pinching broke the apical dominance of the crop which led to utilization of energy for lateral branching and prevention of flower primordial development, hence, delaying the days to flower opening. While T_7_ and T_4_ showed early flowering opening, control required a longer duration (Table 3). This could be due to the difference in nutrient content of the treatments. The observations of Sheoran et al. (2022) were in consonance with the present findings, who reported opening of flower at 57.87 days with pinching performed at 2 weeks after transplanting. It was observed that there was a delay in the number of days taken from planting till full bloom (Joshna and Pal, 2015). This might be attributed to the performance of double pinching which delayed the reproductive growth. However, the number of days taken by T_4_ and T_7_ was comparatively less than control, whose reproductive growth was delayed the most. The variation in the treatments regarding the number of days might be because of the difference in the nutrient content of the potting media. Similarly, the flowering duration inT_7_ and T_4_displayed the same mean values followed by control (Table 3). The results were akin to those reported by Srinivas and Rajasekharam (2020). The number of days from opening to fading of individual flower was observed to be 49.63 days in both the treatments. Environmental factors like rainfall and hormonal regulation might have affected the early fading of flower. The results are in accordance with Yadav et al. (2018). Similarly, flower bud diameter was high in both T_7_ and T_4_. But the largest diameter and fresh weight of flower was recorded in T_7_. This might be due to superiority of T_7_ in terms of nutrient content. Minimum bud diameter registered in control showed significant effect of the absence of termite mound soil on the bud diameter. The diameter of flowers and fresh weight might have been affected by the pinching as it diversifies energy to the lateral branches. However significant differences among the treatments might be due to the difference in the nutrient availability and chemico-physical properties of potting media. The control treatment produced the highest number of flowers followed by T_7_ with T_4_ having the least number of flowers. More number of flowers correlated with smaller flower size and weight. Similar works were carried out by Poudel et al. (2017) and Sheoran et al. (2022). Increase in the number of flowers might have been the reason for lesser fresh weight and diameter as there was lesser quantity of supply of food reserve to each of the individual flowers. As regards to yield, T_7_ expressed the highest yield followed by T_4_ and control (Table 3). It was mostly influenced by the number of flowers, fresh weight and flower diameter.

The highest B:C ratio as a potted plant and as a potting media was observed in T_7_ to be 1.10 and 2.52, respectively. This is because of the highest net return in T_7_ due to its best performance in growth parameters. From the observations of Table 3, it can be inferred that the potting media: T_7_ was found to be significantly superior in terms of plant spread, leaf area index, fresh weight of flower, flower diameter and yield of flower but observed to be statistically at par with T_4_ in terms of plant height, number of branches, number of leaves, stem diameter, number of roots, root length, root diameter, days to visibility of first flower bud, days to opening of flower, days to full bloom, flowering duration on plant, days from opening to fading of individual flower, number of flowers per plant and flower bud diameter. However, the control was found to be significantly superior in terms of number of flowers per plant. Economically, T_7_ was also found to be another viable choice as compared to the other treatments. Hence, we recommend T_7_ potting media to be used for commercial purposes for growing floriculture crops.

## Conclusion

From the current research findings, one is assured of the multiple benefits that can be achieved by using termite mound soil as a potting media. Incorporating termite mound soil into agricultural systems represents a sustainable and eco-friendly approach to farming, particularly in regions with limited access to external inputs and low soil fertility. By harnessing the benefits of termite mound soil, farmers can improve soil fertility, enhance crop productivity, and reduce excessive dependence on synthetic pesticides. The rich nutrient content of termite mound soil based potting media is a true example of conversion of “biowaste to biowealth.” Being a low-cost technology, the invention can easily be explored by the farmers engaged in floriculture and ornamental nursery, peri-urban agriculture and rooftop gardening.

## Data availability statement

The original contributions presented in the study are included in the article/Supplementary material, further inquiries can be directed to the corresponding authors.

## Author contributions

SK: Writing – original draft, Writing – review & editing. BB: Writing – original draft, Writing – review & editing. SB: Writing – original draft, Writing – review & editing. BG: Writing – original draft, Writing – review & editing. PH: Writing – original draft, Writing – review & editing. SG: Writing – original draft, Writing – review & editing. KB: Writing – original draft, Writing – review & editing. PD: Writing – original draft, Writing – review & editing.

## References

[ref1] Ackerman I. L. Teixeira W. G. Riha S. J. Lehmann J. Fernandes E. C. M. (2007). The impact of mound-building termites on surface soil properties in a secondary forest of Central Amazonia. Appl. Soil Ecol. 37, 267–276. doi: 10.1016/j.apsoil.2007.08.005

[ref2] Adebajo S. O. Akintokun P. O. Ezaka E. Ojo A. E. Olannye D. U. Ayodeji O. D. (2021). Use of termitarium soil as a viable source for biofertilizer and biocontrol. Bull. Natl. Res. Cent. 45:100. doi: 10.1186/s42269-021-00560-8

[ref3] Adugna W. T. Lellisa A. Tilahun A. (2016). The impacts of mound-building termites on micronutrients and soil hydraulic properties in parts of Borana lowlands southern Ethiopia. Int. J. Nat. Resour. Ecol. Manag. 1, 32–41. doi: 10.11648/j.ijnrem.20160102.14

[ref4] Aparna D. Reddy M. L. N. Rao A. V. D. D. Bhaskar V. V. Subbaramamma P. Krishna K. U. (2021). Effect of media and hormones on rooting of African marigold stem cuttings in mist chamber. J. Res. Acharya N G Ranga Agric. University. 49, 29–44.

[ref5] Apori S. O. Murongo M. Hanyabui E. Atiah K. Byalebeka J. (2020). Potential of termite mounds and its surrounding soils as soil amendments in smallholder farms in Central Uganda. BMC. Res. Notes 13:397. doi: 10.1186/s13104-020-05236-6 32854759 PMC7457291

[ref6] Ashraf A. Qureshi N. A. Shaheen N. Iqbal A. Fatima H. Afzal M.. (2020). Termiticidal and protozocidal potentials of eight tropical plant extracts evaluated against Odontotermes obesus Rambur (Blattodea; Termitidae) and Heterotermes indicola Wasmann (Blattodea; Rhinotermitidae). Pol. J. Environ. Stud. 29, 3493–3507. doi: 10.15244/pjoes/116105

[ref7] Bachha B. Sahoo S. Misha S. S. Kusum A. (2022). Physicochemical properties and biochemical activities of termitaria soil of Odontotermes spp. and surrounding soil in Sambalpur district, Odisha. India. J. Entomol. Zool. Stud. 10, 124–128. doi: 10.22271/j.ento.2022.v10.i2b.8978

[ref8] Baligar V. C. Staley T. E. Wright R. J. (1991). Enzyme activities in Appalachian soils: 2. Urease. Commun. Soil Sci. Plant Anal. 22, 315–322. doi: 10.1080/00103629109368418

[ref9] Bama P. S. Ravindran A. D. (2018). Isolation and characterization of biological growth promoters from gut region of subterranean termites. Asian J. Res. Soc. Sci. Humanit. 8, 1–5. doi: 10.5958/2249-7315.2018.00120.X

[ref10] Bhairavi K. S. Bhattacharyya B. Devi G. Bhagawati S. Das P. P. G. Devi E. B.. (2021). Evaluation of two native entomopathogenic nematodes against Odontotermesobesus (Rambur) (Isoptera: Termitidae) and Agrotisipsilon (Hufnagel) (Lepidoptera: Noctuidae). Egypt. J. Biol. Pest Control. 31:111. doi: 10.1186/s41938-021-00457-8

[ref11] Bhattacharyya B. Misha H. Gogoi D. Bhagawati S. (2014). Management of termite in preserved setts of sugarcane (Saccharum officinarum) with microbes. Curr. Adv. Agric. Sci. 6, 176–179. doi: 10.5958/2394-4471.2014.00014.8

[ref12] Biswas S. Deka K. (2019). A study on the diversity of termites with reference to their morphometrics and mound construction in Tezpur of Sonitpur district, Assam. India. Int. J. Basic Appl. Biol. 6, 198–203.

[ref13] Brossard M. Lopez-Hernandez D. Lepage M. Leprun J. C. (2007). Nutrient storage in soils and nests of mound-building Trinervitermes termites in Central Burkina Faso: consequences for soil fertility. Biol. Fertil. Soils 43, 437–447. doi: 10.1007/s00374-006-0121-6

[ref14] Casida L. E. Klein D. A. Santoro R. (1964). Soil dehydrogenase activity. Soil Sci. 98, 371–376. doi: 10.1097/00010694-196412000-00004, PMID: 38881156

[ref15] Cheik S. Harit A. Bottinelli N. Jouquet P. (2022). Bioturbation by dung beetles and termites. Do they similarly impact soil and hydraulic properties? Pedobiologia 95:150845. doi: 10.1016/j.pedobi.2022.150845

[ref16] Chisanga K. Mbega E. R. Ndakidemi P. A. (2020). Prospects of using termite mound soil organic amendment for enhancing soil nutrition in southern Africa. Plan. Theory 9:649. doi: 10.3390/plants9050649, PMID: 32443902 PMC7284692

[ref17] Dhembare A. J. (2013). Physico-chemical properties of termite mound soil. Arch. Appl. Sci. Res. 5, 123–126.

[ref18] Dhembare A. J. (2014). Impact of termite activity on physico-chemical properties of mound soil. Cent. Eur. J. Exp. Biol. 3, 25–28.

[ref19] Eze P. N. Kokwe A. Eze J. U. (2020). Advances in nanoscale study of organomineral complexes of termite mounds and associated soils: a systematic review. Appl. Environ. Soil Sci. 2020, 1–9. doi: 10.1155/2020/8087273

[ref20] Garba M. Cornelis W. Steppe K. (2011). Effect of termite mound material on the physical properties of sandy soil and on the growth characteristics of tomato (Solanum lycopersicum L.) in semi-arid Niger. Plant Soil 338, 451–466. doi: 10.1007/s11104-010-0558-0

[ref21] Haldar S. Sengupta S. (2015). Plant-microbe cross-talk in the rhizosphere: insight and biotechnological potential. Open Microbiol J. 9, 1–7. doi: 10.2174/1874285801509010001, PMID: 25926899 PMC4406998

[ref22] Hamarashid N. H. Othman M. A. Hussain M. H. (2010). Effects of soil texture on chemical compositions, microbial populations and carbon mineralization in soil. Egypt. J. Exp. Biol. 6, 59–64.

[ref23] Harit A. Jouquet P. (2021). Origin and dynamics of termite mound soils in southern India. Asian Soil Res. J. 5, 19–23. doi: 10.9734/ASRJ/2021/v5i430115

[ref24] Harit A. Shanbhag R. Chaudhary E. Cheik S. Jouquet P. (2017). Properties and functional impact of termite sheeting. Biol. Fertil. Soils 53, 743–749. doi: 10.1007/s00374-017-1228-7, PMID: 32610265

[ref25] Ibrahim A. K. Abubakar T. Bappah M. Muhammad Z. (2022). Soil physical and chemical properties of termite mound and their adjacent soil in Kashere Akko local government, Gombe state. Nigeria. Int. J. Agric. Rural Dev. 25, 6450–6456.

[ref26] Issoufou A. A. Maman G. Soumana I. Kaiser D. Konate S. Mahamane S.. (2019). Termite footprints in restored versus degraded agrosystems in south West Niger. Land Degrad. Dev. 31, 500–507. doi: 10.1002/ldr.3466

[ref27] Jackson M. L. (1973). Soil chemical analysis. New Delhi, India: Prentice-Hall of India Pvt Ltd.

[ref28] Jenkinson D. S. Powlson D. S. (1976). The effects of biocidal treatments on metabolism in soil-V: a method for measuring soil biomass. Soil Biol. Biochem. 8, 209–213. doi: 10.1016/0038-0717(76)90005-5

[ref29] Jose S. Maya P. M. (2020). Physico-chemical properties and plant growth analysis in termite mound soil and normal soil. Indian J. Appl. Res. 10. doi: 10.36106/ijar

[ref30] Joshna K. Pal P. (2015). Effect of planting date on growth, development, aerial biomass partitioning and flower productivity of marigold (Tagetes erecta L.) cv. Siracole in indo-gangetic plains of West Bengal. J. Appl. Hortic. 17, 151–154. doi: 10.37855/jah.2015.v17i02.28

[ref31] Jouquet P. Guilleux N. Shanbhag R. R. Subramanian S. (2015). Influence of soil type on the properties of termite mound nests in southern India. Appl. Soil Ecol. 96, 282–287. doi: 10.1016/j.apsoil.2015.08.010

[ref32] Jouquet P. Harit A. Herve V. Moger H. Carrijo T. Donoso D. A.. (2022). The impact of termites on soil sheeting properties is better explained by environmental factors than by their feeding and building strategies. Geoderma 412:115706. doi: 10.1016/j.geoderma.2022.115706

[ref33] Jouquet P. Henry-des-Tureaux T. Bouet C. Labiadh M. Caquineau S. Aroui Boukbida H.. (2021). Bioturbation and soil resistance to wind erosion in southern Tunisia. Geoderma 403:115198. doi: 10.1016/j.geoderma.2021.115198

[ref34] Kandeler E. Gerber H. (1988). Short-term assay of soil urease activity using colorimetric determination of ammonium. Biol. Fertil. Soils 6, 68–72. doi: 10.1007/BF00257924

[ref35] Kartik Jain A. K. (2022). Effect of suitable amendments on the water holding capacity of soils. A review. The Pharma Innovation J. 11, 612–615.

[ref36] Kaur H. Singh J. Singh B. (2021). Importance and prospects of marigold. Just Agric. 2.

[ref37] Keen B. A. Raczkowski H. (1921). The relation between the clay content and certain physical properties of a soil. J. Agric. Sci. 11, 441–449. doi: 10.1017/S0021859600004469, PMID: 28314220

[ref38] Khobragade R. K. Bisen S. Thakur R. S. (2012). Effect of planting distance and pinching on growth, flowering and yield of China aster (Callistephus chinensis) cv. Poornima. Indian J. Agric. Sci. 82, 334–339. doi: 10.56093/ijas.v82i4.16645

[ref39] Lee K. E. Wood T. G. (1971). Termite and soils. Exp. Agric. 8:281. doi: 10.1017/S0014479700005354

[ref40] Li Y. Dong Z. Y. Pan D. Z. Pan C. H. Chen L. H. (2017). Effect of termites on soil pH and its application for termite control in Zhejiang province, China. Sociobiol. 64, 317–326. doi: 10.13102/sociobiology.v64i3.1674

[ref41] Meena Y. Sirohi H. S. Tomar B. S. Kumar S. (2015). Effect of planting time, spacing and pinching on growth and seed yield traits in African marigold (Tagetes erecta L.) cv. Pusa NarangiGainda. Indian J. Agric. Sci. 85, 797–801. doi: 10.56093/ijas.v85i6.49231

[ref42] Miyagawa S. Koyama Y. Kokubo M. Matsushita Y. Adachi Y. Sivilay S.. (2011). Indigenous utilization of termite mounds and their sustainability in a rice growing village of the central plain of Laos. J. Ethnobiol. Ethnomed. 7:24. doi: 10.1186/1746-4269-7-24 21849087 PMC3174111

[ref43] Mullins A. Chouvenc T. Su N. Y. (2021). Soil organic matter is essential for colony growth in subterranean termites. Sci. Rep. 11:21252. doi: 10.1038/s41598-021-00674-z 34711880 PMC8553850

[ref44] Panse V. G. Sukhatme P. V. (1985). Statistical methods for agricultural workers. Indian Council of Agricul. Res. Publications., 87–89.

[ref45] Pant P. Mayengbam S. D. Babita Singh H. (2022). Effect of spacing and pinching on growth, flowering and seed yield traits in African marigold (Tagetes erecta) cultivar Pusa NarangiGainda under semi-arid conditions of Haryana, India. Ecol. Environ. Conserv. 28, 29–S205. doi: 10.53550/EEC.2022.v28i01s.029

[ref46] Poudel S. Regmi R. Pun U. Rijal A. (2017). Influence of spacing and pinching on growth parameters of African marigold cv. Inca Orange-1KS. Proceedings of the Ninth National Horticulture Workshop, May 31–June 1, 345–350

[ref47] Roose-Amsaleg C. Mora P. Harry M. (2005). Physical, chemical and phosphatase activities characteristics in soil-feeding termite nests and tropical rainforest soils. Soil Biol. Biochem. 37, 1910–1917. doi: 10.1016/j.soilbio.2005.02.031

[ref48] Sheoran S. Beniwal B. S. Dalal R. P. S. (2022). Floral and yield attributes of African marigold as influenced by pinching and gibberellic acid in different seasons. Pharma Innovation. 11, 937–946.

[ref49] Singh A. K. Kumar U. Kumar A. (2015). Effect of planting date and spacing on performance of marigold (Tagetes erecta L.) cv. Pusa Narangi under North Bihar agro-ecological conditions. Int. J. For. Crop Improv. 6, 16–20. doi: 10.15740/HAS/IJFCI/6.1/16-20

[ref50] Singh V. Singh A. K. Sisodia A. (2017). Growth and flowering of marigold as influenced by pinching and spraying of nitrogen. Int. J. Curr. Microbiol. Appl. Sci. 6, 2283–2287. doi: 10.20546/ijcmas.2017.607.268

[ref51] Srinivas P. T. Rajasekharam T. (2020). Evaluation of marigold genotypes under tropical conditions of Tirupati. Int. Arch. Appl. Sci. Technol. 11, 85–89.

[ref52] Subbiah B. V. Asija G. L. (1956). A rapid procedure for the estimation of available nitrogen in soils. Curr. Sci. 25, 259–260.

[ref53] Subi S. Sheela A. M. (2020). Microbial activity and cellulose degraders in termite mound soil. Int. J. Curr. Microbiol. Appl. Sci. 9, 2154–2161. doi: 10.20546/ijcmas.2020.907.251

[ref54] Tabatabai M. A. Bremner J. M. (1969). Use of p-nitrophenol phosphate for the assay of soil phosphatase activity. Soil Biol. Biochem. 1, 301–307. doi: 10.1016/0038-0717(69)90012-1, PMID: 24713147

[ref55] Walkley A. J. Black I. A. (1934). Estimation of soil organic carbon by the chomic acid titration method. Soil Sci. 37, 29–38. doi: 10.1097/00010694-193401000-00003

[ref56] Yadav K. S. Pal A. K. Singh A. K. Yadav D. Mauriya S. K. (2018). Effect of different bio-fertilizers on growth and flowering of marigold. J. Pharmacogn. Phytochem. 7, 1548–1550.

[ref57] Zhang J. Yang J. Yao R. Yu S. Li F. Hou X. (2014). The effects of farmyard manure and mulch on soil physical properties in a reclaimed coastal tidal flat salt-affected soil. J. Integr. Agric. 13, 1782–1790. doi: 10.1016/S2095-3119(13)60530-4

