# Supplementary figures and images for: Termite mound soil based potting media: a better approach towards sustainable agriculture
Source: Front Microbiol. 2024 Jul 1;15:1387434. doi: 10.3389/fmicb.2024.1387434 (PMC11246991; doi:10.3389/fmicb.2024.1387434)

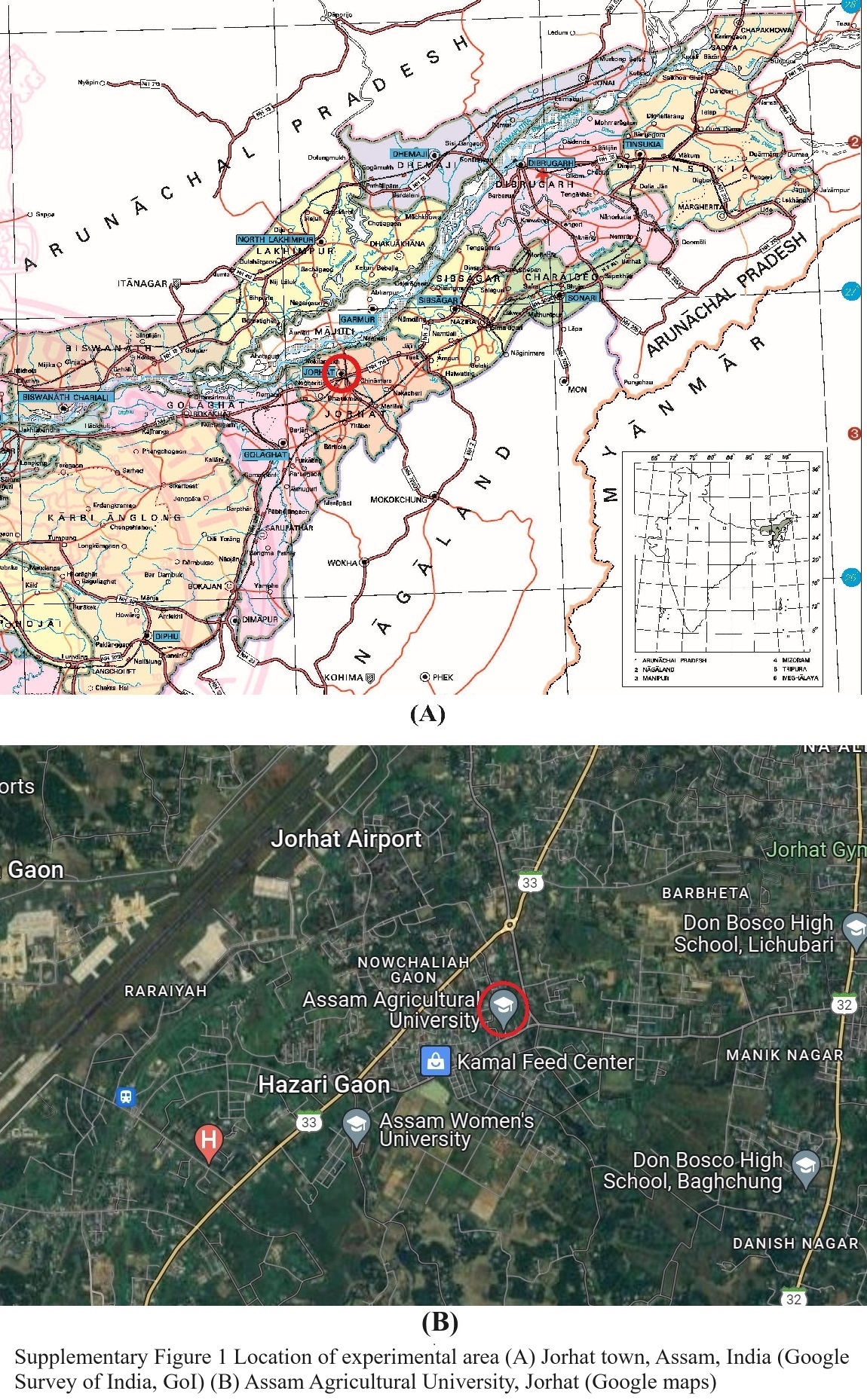

Supplement: Supplementary file 1 [file Image_1.jpg]
